# Genomic analyses reveal FAM84B and the NOTCH pathway are associated with the progression of esophageal squamous cell carcinoma

**DOI:** 10.1186/s13742-015-0107-0

**Published:** 2016-01-11

**Authors:** Caixia Cheng, Heyang Cui, Ling Zhang, Zhiwu Jia, Bin Song, Fang Wang, Yaoping Li, Jing Liu, Pengzhou Kong, Ruyi Shi, Yanghui Bi, Bin Yang, Juan Wang, Zhenxiang Zhao, Yanyan Zhang, Xiaoling Hu, Jie Yang, Chanting He, Zhiping Zhao, Jinfen Wang, Yanfeng Xi, Enwei Xu, Guodong Li, Shiping Guo, Yunqing Chen, Xiaofeng Yang, Xing Chen, Jianfang Liang, Jiansheng Guo, Xiaolong Cheng, Chuangui Wang, Qimin Zhan, Yongping Cui

**Affiliations:** Translational Medicine Research Center, Shanxi Medical University, Taiyuan, Shanxi 030001 China; Key Laboratory of Cellular Physiology, Ministry of Education, Shanxi Medical University, Taiyuan, Shanxi 030001 China; Department of Pathology, First Hospital of Shanxi Medical University, Taiyuan, Shanxi 030001 China; Department of Oncology, First Hospital of Shanxi Medical University, Taiyuan, Shanxi 030001 China; Department of Tumor Surgery, Shanxi Cancer Hospital, Taiyuan, Shanxi 030001 China; Department of General Surgery, First Hospital of Shanxi Medical University, Taiyuan, Shanxi 030001 China; Department of Pathology, Shanxi Cancer Hospital, Taiyuan, Shanxi 030001 China; Department of Urology Surgery, First Hospital of Shanxi Medical University, Taiyuan, Shanxi 030001 China; Department of Endoscopy, Shanxi Provincial People’s Hospital, Taiyuan, Shanxi 030001 China; Institute of Translational Medicine, Shanghai General Hospital, Shanghai Jiao Tong University School of Medicine, Shanghai, 201620 China; Cancer Institute and Cancer Hospital, State Key Laboratory of Molecular Oncology, Chinese Academy of Medical Sciences and Peking Union Medical College, Beijing, 100021 China

**Keywords:** Next-generation sequencing, ESCC, *FAM84B*, NOTCH signaling

## Abstract

**Background:**

Esophageal squamous cell carcinoma (ESCC) is the sixth most lethal cancer worldwide and the fourth most lethal cancer in China. Genomic characterization of tumors, particularly those of different stages, is likely to reveal additional oncogenic mechanisms. Although copy number alterations and somatic point mutations associated with the development of ESCC have been identified by array-based technologies and genome-wide studies, the genomic characterization of ESCCs from different stages of the disease has not been explored. Here, we have performed either whole-genome sequencing or whole-exome sequencing on 51 stage I and 53 stage III ESCC patients to characterize the genomic alterations that occur during the various clinical stages of ESCC, and further validated these changes in 36 atypical hyperplasia samples.

**Results:**

Recurrent somatic amplifications at 8q were found to be enriched in stage I tumors and the deletions of 4p-q and 5q were particularly identified in stage III tumors. In particular, the *FAM84B* gene was amplified and overexpressed in preclinical and ESCC tumors. Knockdown of *FAM84B* in ESCC cell lines significantly reduced *in vitro* cell growth, migration and invasion. Although the cancer-associated genes *TP53*, *PIK3CA*, *CDKN2A* and their pathways showed no significant difference between stage I and stage III tumors, we identified and validated a prevalence of mutations in *NOTCH1* and in the NOTCH pathway that indicate that they are involved in the preclinical and early stages of ESCC.

**Conclusions:**

Our results suggest that *FAM84B* and the NOTCH pathway are involved in the progression of ESCC and may be potential diagnostic targets for ESCC susceptibility.

**Electronic supplementary material:**

The online version of this article (doi:10.1186/s13742-015-0107-0) contains supplementary material, which is available to authorized users.

## Background

Esophageal squamous cell carcinoma (ESCC) is the eighth most common and the sixth most lethal cancer worldwide with approximately 70 % of global esophageal cancer occurring in China [1]. ESCC is more prevalent in non-Caucasian populations, with the highest incidence in the Taihang Mountain region of North-Central China [1], and known risk factors include environmental factors such as dietary habits (e.g. the consumption of hot food and betelnut chewing), family history, alcohol abuse and tobacco smoking [2–4]. ESCC has a highly variable clinical outcome, with an excellent prognosis for stage I and II tumors but a poor outcome for later stage tumors [1]. Currently, three types of treatment are available: surgery, chemotherapy and radiation therapy [5]. Among these, chemoradiotherapy (CRT) is recognized as one of the most effective treatments for ESCC [6]. However, as with other treatments, the clinical response to CRT varies between individuals, and this has a major influence on clinical care outcomes [7, 8]. The 5-year survival rate ranges from 10 % to 25 %, and is highly dependent on tumor stage [9].

The genomic characterization of ESCC tumors, particularly those from different stages of the disease, is likely to reveal underlying oncogenic mechanisms and new cancer-associated genes [10]. ESCC initiates from atypical hyperplasia and progresses to carcinoma *in situ* and then invasive carcinoma [11]. Biomarkers identified from atypical hyperplasia or from early-stage tumors may lead to the development of new diagnostic, prognostic, therapeutic and prevention strategies. Analyses of somatic copy number alterations (SCNAs) using array-based technologies have identified frequently altered regions such as 3q26 [12], 11q13.3 [13] and 8q24.3 [14], and exome-wide investigations have revealed point mutations in the well-known cancer-associated genes *TP53*, *PIK3CA*, *CDKN2A* and novel genes *ZNF750*, *FAT1*, *FAT2* and *FAM135B* [15–17]. However, the genetic or mechanistic alterations related to the progression of ESCC have not been fully elucidated. Thus, there is an urgent need to elucidate the genomic alterations and molecular events associated with the various ESCC stages to enhance our understanding of these tumors, aid in early diagnosis, identify therapeutic targets and develop prevention strategies.

In this study, we used whole-genome sequencing (WGS) of 14, whole-exome sequencing (WES) of 90 and deep target capture sequencing (TCS) of 96 ESCC tumors and adjacent normal tissue from patients recruited from the Taihang Mountain region in North-Central China. This cohort includes 51 stage I and 53 stage III cases from the Han Chinese population who live in the Shanxi and Henan provinces. The genetic alterations that we identified were further validated through next-generation sequencing (NGS) and their association with a specific clinical stage was confirmed by comparison with 36 atypical hyperplasia (i.e. premalignant) tissues.

## Data description

Genomic DNAs were extracted from 3 stage IA, 48 stage IB, 31 stage IIIA, 17 stage IIIB, 5 stage IIIC tumors and matched normal tissues (Additional file 1: Table S1) [18]. WGS libraries (500 bp inserts) and WES libraries (150–200 bp inserts) were constructed and sequenced on an Illumina HiSeq 2000 sequencer using 90 bp paired-end reads. TCS was performed following a protocol similar to WES (see Methods). Sequencing reads from the Illumina HiSeq 2000 sequencer were processed by Illumina software and passed to the in-house pipeline to determine somatic point mutations, indels, and copy number variations. A significance analysis method, MutSigCV, was used to identify significantly mutated genes (SMGs). Fluorescence *in situ* hybridization (FISH), qPCR copy number analyses or targeted PCR-Sanger sequencing was used to validate stage-associated genetic alterations in 36 atypical hyperplasia tissues. To introduce the gene that encodes the family with sequence similarity 84, member B protein (*FAM84B*; also known as *NSE2*) or scrambled control siRNAs into ESCC cells, we used the pLKO.1 virus according to the manufacturer’s instructions. Western blotting was carried out using standard protocols [19] and tissue microarray resourced immunohistochemistry analysis and functional assays were performed as specified in the Methods section.

## Results

### The genome landscape of ESCC and its stage-associated variations

We performed genome sequencing on DNA from 104 ESCC tumors and matched adjacent normal tissues, including 51 stage I and 53 stage III cases (Additional file 1: Table S1) [18]. Five stage I and nine stage III tumors underwent WGS (median coverage of 65×); 46 stage I and 44 stage III tumors underwent WES (median coverage of 132×; Additional file 2: Figure S1). The average number was 3.9 coding mutations/Mb for WGS and 2.4 non-silent mutations/Mb for WES (Additional file 3: Table S2) [18]. Candidate non-silent mutations identified from 48 stage I and 48 stage III tumors were selected for TCS (at least 365×; Additional file 3: Table S2C) [18]. The validation rates were 97.8 % for identified single-nucleotide variations (SNVs) and 58 % for indels. Consequently, we obtained 10,330 somatic point mutations in total: of these, 65 % resulted in missense changes and 6 % resulted in nonsense changes. There were 184 alterations of splice sites and 90 small indels: of the indels, 84 % introduced frameshifts and 16 % were in-frame (Additional file 4: Table S3 and Additional file 5: Figure S2B). We found 73 (range 24–189) non-silent mutations per tumor in this cohort, and this rate is in line with published rates (Additional file 5: Figure S2C) [15–17, 20], underscoring the representative nature of our analysis.

**Fig. 1:**
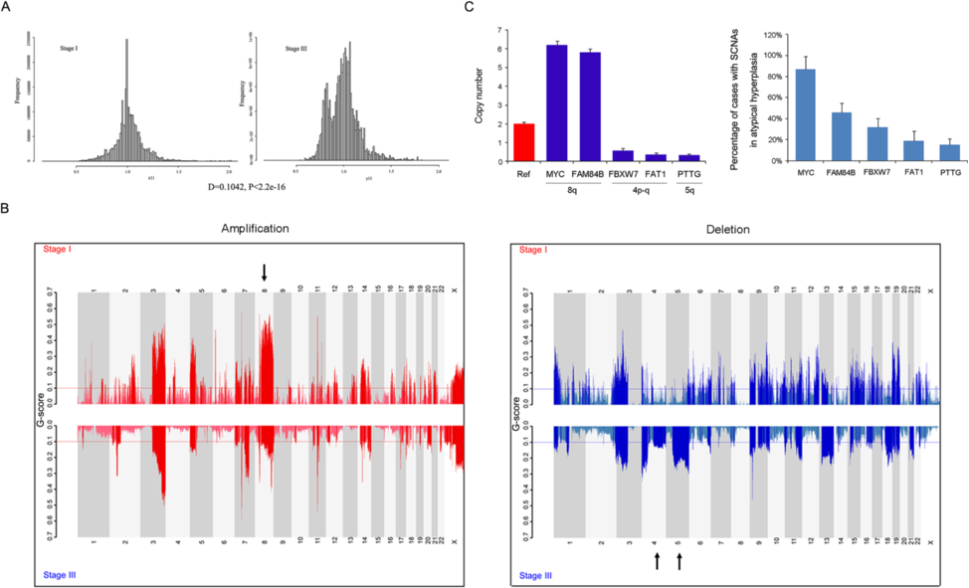
Comparison of copy-number alterations between stage I and stage III ESCC tumors. **a** Comparison of broad structural genome alterations between stage I and stage III ESCC tumors. Analysis is based on absolute copy numbers. Whole-genome sequencing-based analyses reveal that stage III harbor markedly more SCNAs than stage I tumors (*p* < 2.2 × 10^−16^). **b** Significant, focally amplified (red, left panel) and deleted (blue, right panel) regions of stage I (upper) and stage III (lower) are plotted along the genome. The line represents a G-score of 0.1. The black arrows show significantly amplified regions in stage I tumors or deleted regions in stage III tumors. **c** Left panel: Copy number assay by qPCR of candidate genes located in regions significantly associated with various tumor stages in 36 atypical hyperplasia tissues (blue). The RNase P gene was used as reference normal (red). Data are mean ± SD. All assays were performed in triplicate. Right panel: The percentage of cases with copy-number amplification for *MYC*, *FAM84B* or copy-number loss for *FBXW7*, *FAT1* and *PTTG* in 36 atypical hyperplasia tissues

Next, to identify possible differences in the overall genomic architecture between stage I and stage III tumors, we compared the spectrum of mutations and broad SCNAs in these two sets. We observed no clear difference between stage I and stage III tumors in terms of genome-wide somatic SNV counts, even when the groups were subdivided into stage IA, IB, IIIA, IIIB and IIIC (Additional file 6: Figure S3). However, we observed marked differences in the stage-associated pattern of SCNAs. Stage I tumors harbored fewer SCNAs than stage III tumors (*p* < 2.2 × 10^−16^, Fig. 1a) but there was no correlation within sub-stage IIIA, IIIB or IIIC (Additional file 6: Figure S3B). Genomic identification of significant targets in cancer (GISTIC) [21] analysis in the WGS set yielded universal deletions affecting 4p, 11p, 16p, 19p and 19q, and frequent gains of 3q, 5p, 7p, 7q, 8p, 8q, 12p, 14q, 18p, 20q, 21q, Xp and Xq (Additional file 6: Figure S3C). In particular, recurrent somatic amplifications at 8q (containing *MYC* and *FAM84B*) [22] were found to be enriched in stage I tumors; the deletions of 4p-q (containing *VEGFC*, *FBXW7* and *FAT1*) [23, 24] and 5q (containing *PTTG* and *MAML*1) [25, 26] were particularly identified in stage III tumors (*p* < 0.05, Fisher’s exact test, Fig. 1b), suggesting that these alterations are associated with stage progression. Furthermore, copy-number analyses verified the amplifications of candidate genes located within these significantly altered regions in 36 atypical hyperplasia tissues (Fig. 1c). Thus, although stage I and stage III tumors of ESCC are genomically similar, our results reveal that the copy-number variations exhibit a pattern that is associated with the clinical stage of the tumor.

**Fig. 2:**
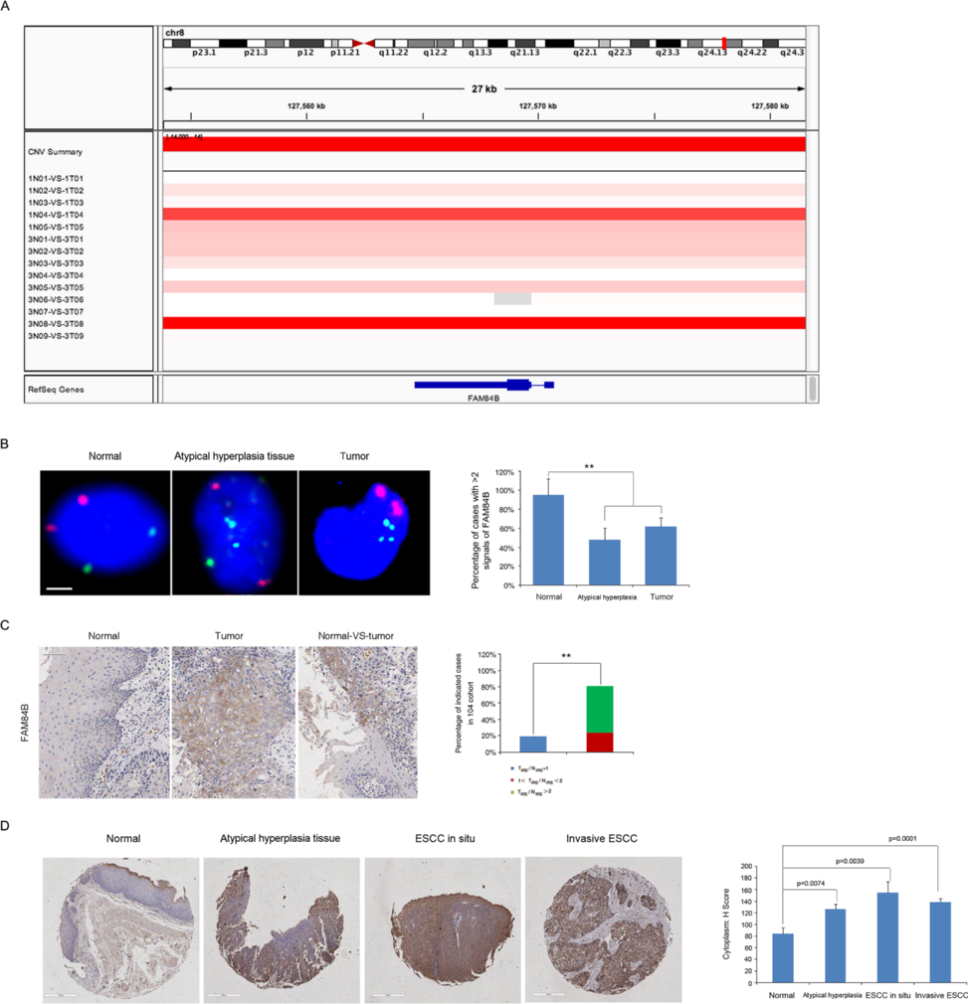
*FAM84B* is amplified and overexpressed in ESCC tumors. **a** Focally amplified (red) region containing *FAM84B* of the WGS set viewed by IGV is plotted along the chromosome. **b** Representative immunofluorescence images show signals produced from FISH analyses using probes specific to chromosome 2 (red) and *FAM84B* (green) in normal, preclinical atypical hyperplasia tissue and an ESCC sample. Scale bars, 5 μm. The bar graph (right panel) shows the percentage of indicated cases with more than two *FAM84B* signals in each group. **c** *FAM84B* is highly expressed in ESCC tumors. Representative immunohistochemistry images show *FAM84B* expression in ESCC tumors and matched normal tissues. The right image shows *FAM84B* expression in one slide with tumor cells and adjacent normal cells. The bar graph (right panel) shows the percentage of indicated cases with varying *FAM84B* expression levels in the 104 patient cohort. *FAM84B* expression level was based on subjective assessment of immunohistochemical staining intensity (see Online Methods). Scale bars, 400 μm. ***p < 0.001. **d** Left panel: Representative images displaying cytoplasmic positivity of *FAM84B* in normal esophagus tissue, atypical hyperplasia tissue, ESCC *in situ* and invasive ESCC tissues from large-scale tissue microarraays (OD-CT-DgEso01-001, Shanghai Outdo Biotech Detail information of cases was shown in Additional file 16: Table S9). Right panel: Cytoplasm expression significantly increases in atypical hyperplasia tissues, ESCC *in situ* and invasive ESCC tissues compared with that of normal esophagus tissues based on a judgment of immunohistochemistry staining intensity (*χ* ^2^-test). The normal esophagus tissues include 10 from the OD-CT-DgEso01-001 array and 104 matched normal esophagus tissues from our sequenced cohort

**Fig. 3:**
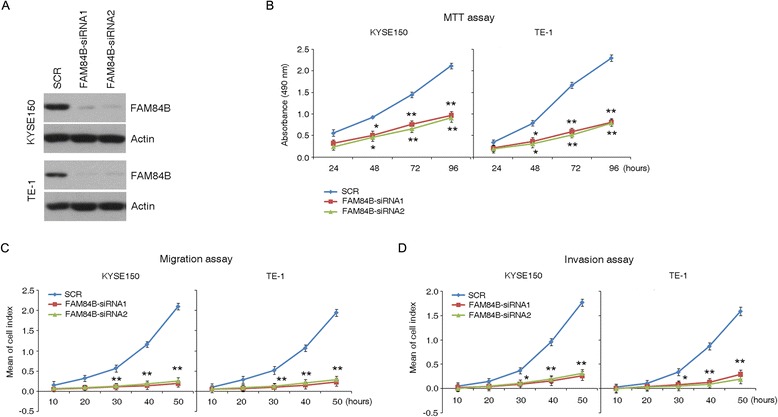
*FAM84B* knockdown dramatically inhibited cell proliferation, cell migration and invasion. **a** The knockdown efficiency of *FAM84B* was verified by western blotting analysis. Actin was used as a loading control. **b** MTT growth assay shows an increase in proliferation following knockdown of *FAM84B* in KYSE150 and TE-1 cells. Data represent the mean ± SD; At least three independent experiments were performed; each experiment was performed in triplicate. Statistical analysis was done using a two-sided *t*-test. *p < 0.05, **p < 0.01. **c**,**d** *In vitro* migration/invasion assay with three experimental replicates revealed that knockdown of *FAM84B* significantly promotes cell migration and invasion in KYSE150 and TE-1 cells. SCR indicates non-specific control siRNA. Each experiment was performed in triplicate; data are mean ± SD. *p < 0.05; **p < 0.01

We also applied a modified GISTIC method to profile genome segments with copy number variations in the 14 tumors analyzed by WGS, which revealed 126 significantly altered regions (Additional file 7: Figure S4). Moreover, to identify genes affected by recurrent SCNAs, we manually inspected the 126 significantly altered regions using the Integrative Genomics Viewer (IGV). This approach identified recurrent focal CNAs, including one of the most amplified regions, 8q24.13-q24.21, which contains *FAM84B* (Additional file 8: Table S4) [27]. Amplification of this gene was found in 44 % (46 out of 104) of patients (Fig. 2a and Additional file 9: Figure S5) and this was further validated by fluorescence *in situ* hybridization (FISH) in atypical hyperplasia tissues and ESCC tumors (Fig. 2b). *FAM84B* is involved in the formation of DNA-repair complexes and little is known about its function in human cancers [27]. In our cohort of 104 patients, this gene was markedly highly expressed in 57 % of cases (the immunoreactivity score for *FAM84B* expression in tumors was at least double that of matched normal tissue, T_IRS_/N_IRS_ > 2, Fig. 2c). To verify the specific abundance of this gene during the initiation of ESCC, we used immunohistochemistry on tissue microarray-sourced preclinical and ESCC samples. We observed a marked relative increase in *FAM84B* expression in atypical hyperplasia samples compared with that of normal tissues (Fig. 2d). In addition, we knocked down *FAM84B* in KYSE150 and TE-1 cells that have high levels of endogenous *FAM84B* and observed that *FAM84B* depletion attenuated cell growth, migration and invasion (Fig. 3). This finding suggests that *FAM84B* amplification and the resultant increased levels of *FAM84B* protein are associated with progression from normal squamous epithelium to ESCC and may be a potential diagnostic marker for susceptibility to ESCC.

### Significantly mutated genes

For most cancer types, genomic landscapes consist of many ‘hills’ (corresponding to genes that are altered in a low percentage of the tumors) and a small number of ‘mountains’ (genes that are altered in a higher percentage of tumors) [28]. This also holds true for ESCC, as revealed by our cohort (Additional file 10: Figure S6; Additional file 11: Table S5) [18]. Only 14 genes (*TP53*, *TTN*, *MUC16*, *NOTCH1*, *FAT1*, *PIK3CA*, *CSMD3*, *PCLO*, *LRPIB*, *MLL2*, *EP300*, *SYNE1*, *SPTA1* and *PKHD1L1*) contained somatic mutations that were detected in at least 10 % of the samples whereas 85 genes were mutated in 5-10 % of the tumors. The remaining 1,309 genes were altered in 2–5 % of the cohort of 104.

**Fig. 4:**
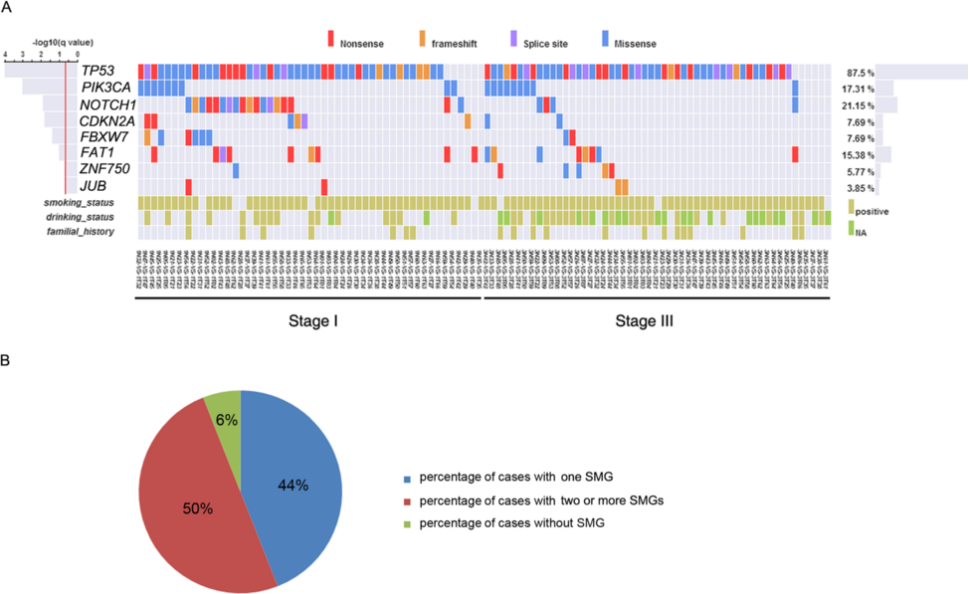
A view of the genome landscape of ESCCs at various stages. **a** Stage-distributions of candidate driver mutations identified by MutSigCV significance analysis. The type of each mutation is shown for every sample, including the gene-specific total number of mutated samples (right); mutation subtypes are denoted by color. If multiple mutations were found in a gene in a single sample, only one is shown. The significance of the mutations in each gene is shown to the left by the false discovery rate (FDR) q value. The lower bars indicate smoking/drinking status and family history, respectively. The full list of mutated genes is given in Additional file 8: Table S4. **b** Percentage of ESCC patients harboring one or two SMGs in the 104 cohort. The ESCC patients harboring none of the identified SMGs are also shown

We applied MutSigCV to identify significantly mutated genes (SMGs) associated with ESCC. This analysis led to the identification of eight SMGs with q < 0.2 (Additional file 12: Table S6A). In general, the number of SMGs in an individual cancer ranges from zero to four. Of the 104 tumors that we comprehensively characterized, 50 % (52 out of 104 cases) displayed mutations in two or more SMGs, 44 % (46 out of 104 cases) of cases harbored alterations in one SMG, and six cases did not have mutations in any of the SMGs. We observed 21 different combinations of SMGs and so there seems to be substantial variation in the drivers of oncogenesis. Four of the eight SMGs (*TP53*, *NOTCH1*, *PIK3CA* and *FAT1*) were mutated in more than 10 % of cases. Collectively, these contributed 86 % (158 of 184) of the non-silent mutations. The other four SMGs, each contributing relatively infrequently, were responsible for the remaining 14 % (26 of 184) of the non-silent mutations (Fig. 4).

### Identical mutations suggest stage-associated NOTCH pathway alterations

**Fig. 5:**
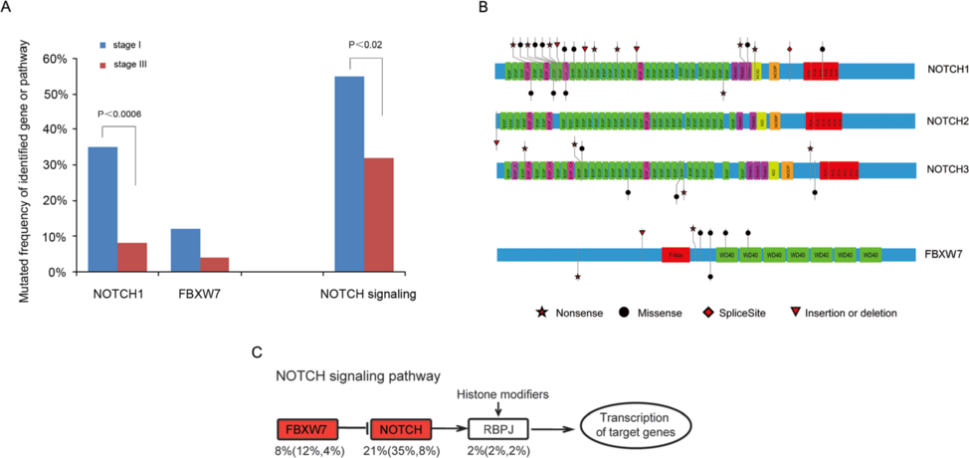
Identification of SMGs and pathways associated with early development of ESCC. **a** Comparison of stage I and stage III tumors. The identified SMGs and top-ranked pathway predominant in stage I of ESCC are shown. Differences in SMGs and altered pathways between stage I and stage III samples were statistically tested by Fisher’s exact test. **b** Schematic representation of the domain structure of the NOTCH family and *FBXW7*, and the location of somatic mutations identified in ESCC tumors. The types and relative positions of confirmed somatic mutations are shown in the transcripts of identified genes using the following symbols: stars, nonsense mutations; circles, missense mutations; diamond, mutations at splice sites; triangles, small insertion or deletion. The upper symbols represent mutations identified in stage I and the lower ones represent mutations identified in stage III tumors. **c** The main mutations involved in the NOTCH signaling pathway in this cohort. The overall percentage of patients carrying these specific mutations is given and this is also broken down into stage I and stage III tumors

To discern the SMGs and pathways relevant to tumorigenic capacity and tumor progression in more detail, we investigated the relationship between the prevalence of each SMG and tumor stage and conducted separate pathway analyses for stage I *versus* stage III tumors. Interestingly, a pronounced diversity of *NOTCH1* mutations was observed in 35 % of stage I tumors but in only 8 % of stage III tumors (*p* < 0.0006, Fisher’s test). Likewise, NOTCH signaling, a fundamental signaling system comprising the NOTCH receptor (including *NOTCH1*, *NOTCH2*, *NOTCH3* and *NOTCH4*), Delta and Serrate/Jagged (DSL) ligands and CSL DNA-binding proteins [28], was altered in 55 % of stage I tumors *versus* 32 % of stage III tumors (*p* < 0.02, Fisher’s test, Fig. 5a). This indicates that alteration of the NOTCH pathway may be early event in the development of a subgroup of ESCCs. *NOTCH1* mutations are relatively common in head and neck squamous cell carcinoma (HNSCC), lung SCC and breast cancer, with 5 % to 15 % of tumors harboring protein-coding changes [28–30]. In our cohort, 22 somatic mutations were identified in *NOTCH1* with a mutation frequency of 21 % (Additional file 12: Table S6B). Of the 22 non-silent mutations, eight are nonsense, ten are missense, three are frameshift indels and one is a splicing site mutation, and these are, in general, all predicted to be loss-of-function mutations (Fig. 5b). Notably, we found another six non-silent mutations including four missense and two frameshift indels, with a mutation frequency of 16.7 % in the 36 atypical hyperplasia tissues (Additional file 13: Table S7). Many of these missense mutations occurred at or near important domains such as the ligand-binding domain (EGF repeats). Moreover, the nonsense mutations observed in *NOTCH1* generate a premature stop codon, resulting in a C-terminally truncated *NOTCH1* protein lacking a PEST sequence (a sequence rich in proline, glutamic acid, serine and threonine) that is important for transcription activation. In addition, five out of nine mutations identified in *NOTCH2/3* were truncating, and two stopgains were identified in *RBPJ*, one of the target genes of *NOTCH*. Thus, in contrast to T-cell acute lymphoblastic leukemia, chronic lymphoblastic leukemia and breast cancer, in which *NOTCH1* serves as an oncogene [31], this pattern of mutations suggest that the NOTCH pathway has a tumor suppressing role in ESCC.

Of related interest, 8 % of ESCC tumors (12 % of stage I tumors and 4 % of stage III tumors) harbored mutations in the F-box protein *FBXW7* (Fig. 5a). *FBXW7* is the substrate-recognition subunit of an SCF-type ubiquitin ligase complex that regulates the cell cycle by targeting many proto-oncoproteins and antiapoptotic molecules including *NOTCH1*, *cyclin E*, *c-Myc*, *c-Jun* and *mTOR* for ubiquitin-mediated degradation. It therefore acts as a tumor suppressor protein [32] and has been found to be mutated in various tumors [33, 34]. Of the eight mutations detected in our cohort, two nonsense and one frameshift deletion are inactivating mutations (Fig. 5b, bottom panel). Moreover, most of the mutations occur within the WD40 domain involved in substrate recognition; mutation of this site prevents the recognition of targets such as *NOTCH1* for degradation. Taken together, we identified 31 mutations in NOTCH family genes. Most of these mutations result in truncated protein products or have deleterious effects, suggesting that NOTCH signaling is significantly disrupted in our sample set and may be one of the main mechanisms associated with the development of a subgroup of ESCCs.

In addition to the NOTCH pathway, we found that mutations in genes involved in several major metabolic pathways were significantly enriched in the stage I group. In particular, mutations occurred in genes associated with pyrimidine metabolism (31 % of stage I tumors *versus* 13 % of stage III), glycine/serine/threonine metabolism (16 % *versus* 2 %) and fructose and mannose metabolism (16 % *versus* 2 %). Conversely, the hedgehog (Hh) signaling pathway showed a significantly higher mutation frequency in the late stage III group (*p* < 0.05, Fisher’s test; Additional file 14: Table S8). Thus, despite there being no marked differences between the tumors at various stages at the level of individual genes, such differences seem to exist at the level of pathways. For other frequently or significantly mutated genes, we found no significant correlation between their mutation frequencies and tumor stage.

## Discussion

Clinical screening and surveillance approaches (i.e. upper gastrointestinal endoscopy, barium esophagram, non-endoscopy-based balloon cytology and serology tumor markers etc.) for the early diagnosis of ESCC are currently limited [1]. Hence, biomarkers indicating ESCC pathogenic processes are urgently required to diagnose and facilitate early intervention. Initial genomic reports have described whole-genome and -exome sequencing for ESCC patients [15–17]. Although informative, these studies did not reveal the genomic differences between tumors from various clinical stages.

In this study, we report the genomic characterization of different stages of ESCC based on either WGS or WES of 104 ESCC patients. We show that amplifications of 8q and deletions of 4p-q and 5q may be associated with the early stages of ESCC. Moreover, we identify *FAM84B* as a novel ESCC-associated gene*.* Further functional and clinical analyses strongly indicate that *FAM84B* (located at 8q24.13-q24.21), which is highly expressed in dysplasia and ESCC patients but not normal esophagus tissues, may contribute to oncogenesis in ESCC and that targeting *FAM84B* may be a promising strategy for the diagnosis of susceptibility and the early stages of ESCC.

Similar to many other cancers, particularly esophageal adenocarcinoma (EAC) [20], the well-defined cancer-associated genes such as *TP53*, *PIK3CA* and *CDKN2A* were also identified as SMGs in ESCC, providing evidence of a common dysfunction in cell-cycle control and apoptotic signaling. Importantly, the analysis of ESCC genomes reveals that mechanisms previously suspected to have a role in the biology of ESCC [15–17, 35] but not in EAC [19] (for example, *NOTCH1* and *FBXW7*) are indeed involved in ESCC development (Additional file 15: Figure S7). Moreover, genomic characterization of different stages of ESCC tumors led us to identify dysregulated *NOTCH1* and NOTCH signaling predominantly in stage I tumors, indicating the involvement of this gene and its pathway in the early development of ESCC. Thus, the prevalence of *NOTCH1* provides a potential biomarker to detect ESCC in its early stages. NOTCH signaling has long been known to function in developmental processes and in regulating the self-renewal of tissues [36, 37]. Whereas activating mutations in *NOTCH1* have been identified in T-cell acute lymphoblastic leukemia and breast cancer [38–40], a tumor suppressor role for NOTCH signaling has been suggested in tumor types such as chronic myelomonocytic leukemia (CMML) [41], HNSCC [29] and lung SCC [42]. The spectrum of mutations involving the NOTCH pathway in our cohort is consistent with it having a tumor suppressor role in ESCC rather than an oncogenic function. It is important to note that although *NOTCH1* has been reported to be a poor survival marker in human ESCC [43], the correlation between genotype and the expression level of *NOTCH1* remains ambiguous.

## Conclusion

In summary, we used the genomics data described above, together with a large-scale tissue platform, to pinpoint molecular features linked to the early occurrence of ESCC. We provide genetic and functional evidence suggesting that overexpression of *FAM84B* is related to the progression of ESCC and may be a potential diagnostic and/or therapeutic target. We also identify the genomic aberrations that frequently alter the NOTCH pathway in stage I ESCC tumors and which may be useful for predicting early ESCC onset. Collectively, our results highlight the substantial stage-associated genetic diversity underlying ESCC and facilitate the understanding of the molecular defects that lead to early disease onset, which may ultimately provide a set of potential targets for its early diagnosis and prevention.

## Methods

### Samples and clinical data

We recruited tumor samples and adjacent normal tissues from 104 ESCC patients from the Han Chinese population who live in the Shanxi and Henan provinces, Taihang Mountain, North-Central China. All samples were obtained before treatment according to the guidelines of the local ethical committees (IRB of Shanxi Medical University, Approval No. 2009029, and the Ethics Committee of Henan Cancer Hospital, Approval No. 2009xjs12). This study was approved by the ethical committee of the Shanxi and Henan, China. All ESCC cases collected for this study were staged using the American Joint Committee on Cancer (AJCC) Cancer Staging Standards, 7th Edition (2010). This cohort includes 51 cases of stage I tumors and 53 cases of stage III tumors. Different subsets of patients were assayed on each platform: 14 tumors and matched normal samples from 14 patients, including 5 cases of stage I and 9 cases of stage III, had WGS data available (65×); 90 samples from 46 cases of stage I and 44 cases of stage III, had WES data available (132×); 96 of the 104 samples including 48 cases of stage I and 48 cases of stage III had TCS data available (365×) [18]. A detailed description of the clinical characteristics of this cohort is shown in Additional file 1: Table S1. A summary of the next-generation sequencing analyses in this study is presented in Additional file 2: Figure S1.

### Sequencing

For WGS, genomic DNA extracted from 5 stage I tumors, 9 stage III tumors and matched normal tissues were randomly fragmented and purified. The WGS libraries were constructed and subjected to WGS on an Illumina HiSeq 2000. At least 65× target depth and 30-fold haploid coverage for tumors and normal samples were achieved in all samples. A detailed description is presented in Additional file 3: Table S2.

For WES, the qualified genomic DNAs from 46 tumors from stage I, 44 tumors from stage III and matched normal tissues were randomly fragmented, amplified by ligation-mediated PCR (LM-PCR), purified and hybridized to the NimbleGen SeqCap EZ exome (44 M, Roche Company, Indianapolis, IN, USA) array for enrichment. Each captured library was subjected to an Illumina HiSeq 2000 platform for high-throughput sequencing. The mean coverage achieved was 130× in the tumor and 133× in the normal tissues. A detailed description is presented in Additional file 3: Table S2B. The Agilent SureSelect in Solution is described elsewhere [44].

### Illumina sequencing analysis pipeline

For detection of somatic point mutations, sequencing reads from an Illumina HiSeq 2000 sequencer were aligned to the human reference genome (hg19) sequence using Burrows-Wheeler Aligner (BWA) [45]. After removing duplicated reads (redundant information produced by PCR) using the SAMtools, an in-house cancer sequencing analysis pipeline (CSAP) was used to identify somatic mutations [15, 46]. Several recently published studies have compared the somatic mutation calling tools (e.g. SomaticSniper, Virmid, Strelka, MuTect and VarScan2) and found that VarScan2 outperformed all other tools in detecting high-quality somatic mutations (high coverage and allele frequency) [47–49]. Therefore, we detected mutations by VarScan2.2.5 with previously determined parameters [50]. High-confident SNVs were annotated with ANNOVAR and used in follow-up analyses [51]. Briefly, we used an in-house pipeline for statistic sequencing error rates, base calling accuracy and ATCG base contents of raw sequencing data. Sequence data per lane needed to meet the following quality controls: *i.*sequencing error rates ≤ 2.5 %; *ii.* base calling accuracy, measured by the Phred quality score (Q score), Q20 ≥ 80 %, Q30 ≥ 75 %; *iii.* GC or AT separation rate ≤ 0.4 %. We applied SOAPnuke to remove adapter and filter low quality reads [52]. Reads were kept if they met the following criteria: *i.* adaptor rate ≤ 10 %; *ii.* N rate of every single read ≤ 10 %; *iii.* low quality base rate (base quality < 5) of every single read ≤ 50 %. After removing reads containing sequencing adaptors and low-quality reads, the high-quality single-end reads were aligned to the NCBI human reference genome (hg19) using BWA by default parameters. Each sample should meet the following criteria: *i.* average sequencing depth of WGS ≥ 30×, average sequencing depth of WES ≥ 100×; *ii.* Mapping rate ≥ 95 %; *iii.* Mismatch rate ≤ 10 %. High-confident somatic SNVs were called when they met the following criteria: *i.* both the tumor and normal samples should be covered sufficiently (≥10×) at the genomic position; *ii.* the variants should be supported by at least 10 % of the total reads in the tumor compared with less than 2 % in normal tissues; *iii.* the variants should be supported by at least three reads in the tumor; *iv*. distance between adjacent somatic SNV distance should be over 10 bp; *v.* mapping qualities of reads supporting mutant alleles in the tumor should be significantly higher than 30 (Wilcoxon rank sum test, *p* < 0.2); *vi.*base qualities of reads supporting mutant alleles in the tumor should be significantly higher than 20 (Wilcoxon rank sum test, *p* < 0.05); *vii.*mutations should not be enriched within 5 bp 5′ or 3′ of read end (Wilcoxon rank sum test, *p* < 0.1); *viii.* the changes of mutant allele frequency between tumor and normal should be statistically significant (Fisher’s exact test, *p* < 0.05); *ix.* reads supporting mutations should not be significantly enriched within either forward or reverse genomic strand (Fisher’s exact test, *p* < 0.0001).

### Detection of small indels

The indel calling step was performed by a GATK SomaticIndelDetector with default parameters [53]. The high-confident indels were identified by an in-house pipeline and further annotated with ANNOVAR [51] as either germline or somatic based on whether any evidence for the event at the same locus was observed in the normal data. High-confident somatic insertions and deletions (indels) were called through the following steps: *i.* candidate somatic indels were predicted with the GATK SomaticIndel Detector with default parameters; *ii.*for each predicted somatic indel, local realignment was performed with combined normal and tumor BAM files; *iii.* high-confident somatic indels were defined after filtering germline events.

### Target capture sequencing

To provide high-confident mutations, non-synonymous mutations identified in the WGS and WES sets were selected for TCS. Briefly, non-synonymous SNVs (6873) and indels (125) in coding regions identified from 96 of the 104 samples were designed on a Nimblegen customized capture array (Roche Company, Indianapolis, IN, USA). Genomic DNAs from 48 stage I tumors, 48 stage III tumors and matched normal tissues were fragmented and libraries were constructed following the same method as the exome-capture experiment. For SNVs and small indels, we tiled ~200 bp targets across the variant of interest, including a minimum buffer of 100 bp in each direction. After library preparation and hybridization, sequencing was performed on an Illumina HiSeq 2000 platform. Each tumor was sequenced to at least a depth of 300× and SNVs were called with the same pipeline except that variant allele frequency was decreased to 5 % to guarantee that low-frequency mutations were retained. Somatic indels were manually inspected across 104 normal samples to remove germline mutations. The mean coverage achieved was 365× in TCS with 354× in tumors and 375× in the normal samples. A detailed description is presented in Additional file 3: Table S2C. Validation lanes were aligned to the reference sequence and BAM files created in the same manner as described above.

### DNA copy number analysis

We performed SegSeq [54], a widely used method to identify copy number variation (CNVs) by comparing a tumor sample with a matched normal sample, to infer somatic CNVs in ESCC genomes based on WGS reads. The resulting copy number segments were mapped to individual genes to determine gene-level copy numbers and copy gain/loss statuses. Copy numbers of ≤ 1.5 were considered to indicate deletions and ≥ 2.5 were considered to be amplifications. To infer recurrently amplified or deleted genomic regions, we re-implemented the GISTIC [21] algorithm using copy numbers in 1 kb windows as markers instead of SNP array probes. G-scores were calculated for genomic and gene-coding regions based on the frequency and amplitude of amplification or deletion of each gene. A significant CNV region was defined as having an amplification or deletion with G-score > 0.1, corresponding to a *p*-value threshold of 0.05 from permutation-derived null distribution.

### Identification of significantly mutated genes

For the identification of significantly mutated genes, we applied a novel analytical methodology, mutation significance with covariates (MutSigCV) [10], to avoid the false-positive findings detected by the standard significance analysis method (MutSig1.0) [55]. MutSigCV corrects for variation by using patient-specific mutation frequency and spectrum and gene-specific background mutation rates, incorporating expression levels and replication time. MutSigCV is freely available for non-commercial use [56].

### Pathway enrichment analysis

We performed the pathway enrichment analysis using the Database for Annotation, Visualization and Integrated Discovery (DAVID) v6.7 by examining the distribution of the non-synonymously mutated genes identified within the KEGG database [57] as described previously [18]. Significantly altered pathways were determined by *p*-values calculated based on hypergeometric distribution with Benjamini correction.

### Cell lines

KYSE150 and TE1 ESCC cell lines that were tested and found to be free of mycoplasma contamination were used in this study. 293 T cells were used as a packaging cell line to produce virus. All cells were grown in DMEM/F12 media at 37 °C in 5 % CO_2_. For the functional analysis of *FAM84B*, the ESCC lines KYSE150 and TE-1 with high endogenous expression levels were used for knockdown experiments.

### Knockdown of *FAM84B*

Knockdown experiments were performed in at least two ESCC lines with high endogenous *FAM84B* expression. Two independent shRNAs were cloned into the pLKO.1-puro vector (Addgene, Cambridge, MA, USA) as described previously [58]. A non-specific targeting shRNA was also cloned into the pLKO.1-puro vector to be used as a scrambled control (SCR). shRNA knockdown efficiency was determined by western blot analysis for *FAM84B* proteins using an anti-*FAM84B* antibody (Proteintech, Chicago, USA). Relative expression was normalized to the β-actin expression level.

### qPCR copy number validated analysis

The copy numbers of the genes of interest were assessed in frozen atypical hyperplasia samples using genomic qPCR (TaqMan, Applied Biosystems, Foster, CA, USA) in triplicate. Prevalidated primers for the relevant genes were obtained from Applied Biosystems (accession numbers Hs02758348_Cn for *MYC*, Hs00655850_Cn for *FAM84B*, Hs01654625_Cn for *VEGFC*, Hs05965356_Cn for *FBXW7* and Hs00703603_Cn for *FAT1*). RNase P (*RPPH1* gene; Life Technologies, Shanghai, China, 4403328) was used as a diploid control. Data were analyzed using the comparative (delta-Ct) Ct method. An inferred copy number of < 0.3 was considered to indicate a homozygous deletion.

### Fluorescence *in situ* hybridization analysis

To evaluate the amplification of *FAM84B*, we performed FISH. Tumor and matched normal tissues of interesting ESCC cases were cut into pieces in PBS, swollen in 65 mmol/L KCl for 5 mins at 37 °C, followed by fixation in cold acetic acid/methanol for 5 mins at 4 °C, then dropped onto slides. For interphase FISH analysis, slides were stained with Cytocell enumeration probes against chromosome 2 or *FAM84B* conjugated with FITC or Cy3.5 (Rainbow Scientific, Windsor, CT, YSA). Probes against chromosome 2 were used as controls. Staining was carried out according to the manufacturer’s protocol. FISH samples were viewed with a fully automated, upright Zeiss Axio-ImagerZ.1 microscope with a × 20 objective and DAPI, FITC and Rhodamine filter cubes. Images were produced using the AxioCamMRm CCD camera and Axiovision version 4.5 software suite. *p*-values were calculated using a two-sample test for equality of proportions with continuity correction.

### Immunohistochemistry and tissue microarray resource

*FAM84B* was immunohistochemically stained with an anti-*FAM84B* antibody (Proteintech, Chicago, USA) as described previously [18]. A human ESCC tissue array (OD-CT-DgEso01-001) purchased from Shanghai Outdo Biotech (Shanghai, China) was used to detect the expression level of *FAM84B*. OD-CT-DgEso01-001 array contains 10 normal esophagus tissues, 22 atypical hyperplasia tissues and 58 ESCC cases (2cores/case; Additional file 16: Table S9). *FAM84B* immunohistochemistries were performed on tissue microarraysas previously described [59], using the polyclonal anti-*FAM84B* antibody (Additional file 17: Figure S8). Briefly, sections were incubated with the specific antibody at a 1:500 dilution for 14 h at 4 °C, followed by detection using the PV8000 (Zhongshan, Beijing, China) and DAB detection kit (Maixin, Fuzhou, China), producing a dark brown precipitate. Slides were counterstained with hematoxylin. All images were captured at × 100. Cytoplasm expression of *FAM84B* was quantified using AperioCytoplasma 2.0 software. Statistic analyses were performed using Graphpad Prism 5.0.

### Immunoblotting

Immunoblotting was performed as previously described [19] using anti-*FAM84B* (Proteintech, Chicago, USA). Antibody binding was detected using horseradish peroxidase-labeled anti-mouse (Sigma, Santa clara,USA) or anti-rabbit (Cell Signaling, Boston,USA) antibodies and chemiluminescence was measured using a LAS4000 device chemiluminescence System (Sagecreation, Beijing, China). Equal protein loading was confirmed with antibodies against β-actin (Transgen, Beijing, China).

### MTT and migration/invasion assays

To assess cell viability, MTT assays were performed as previously described [59]. Each experiment consisted of four replications and at least three independent experiments were carried out. Migration and invasion assays were performed in 16-well CIM plates in an xCELLigence RTCA DP system (ACEA Biosciences, San Diego, USA) using BD matrigel basement membrane matrix for real-time cell migration analysis as described previously [60, 61]. At least three independent experiments were carried out; for each independent experiment, five duplicates were performed for each group.

### Statistical analysis

Experiments were done in triplicate and data were presented as mean ± SD. Student’s *t*-test was used for statistical analysis, and data from more than two groups were analyzed by one-way analysis of variance (ANOVA) in SPSS Statistics 19.0 followed by LSD-*t* test. Results were considered significant when *p* < 0.05.

## Availability of supporting data

The data sets supporting the results of this article are available in the European Genome-Phenome Archive repository at Study accession EGAS00001001487 and Dataset accession EGAD0000100169. Tissue Microarray data and further details on data access are available from the *GigaScience,* GigaDB database [62].

## Additional files

Additional file 1: Table S1. Clinical features of the ESCC patients providing samples for sequencing. (XLSX 22 kb) Additional file 2: Figure S1. The sequencing data processing pipeline and calculation of coverage. **(a)** Sequencing and analytical pipeline for determining somatic mutations in our cohort. **(b)** Fold coverage of whole genome and exome in the sequenced normal and tumor samples in ESCC. The upper panel: whole-genome sequencing set; the lower panel: whole-exome sequencing set; lfet: the box plot depicts the distribution of mean coverage; right: the box plot depicts the distribution of fraction of bases covered by at least 1 reads, 4 reads, 10 reads and 20 reads across the sequencing samples. N, normal samples; T, tumor samples; All samples were calculated with an average estimated tumor content of 40-50 %. (TIFF 4975 kb) Additional file 3: Table S2. Sequencing and mapping statistics of the 104 samples. **(a)** Coverage, mutation, and rearrangement frequencies for individual samples generated by WGS. **(b)** Coverage and mutation data for individual samples generated by WES. **(c)** Validation of somatic mutations via target-captured sequencing. (XLSX 26 kb) Additional file 4: Table S3. Samples and their individual somatic mutations of exonic and splice-site. (XLSX 14 kb) Additional file 5: Figure S2. Overview of mutations in ESCC and comparison with other tumor types. **(a)** Distribution of non-synonymous mutations identified in WES set. The red and blue spots represent mutations of stage I and stage III tumors, respectively. **(b)** The left pie chart shows the distribution of mutant regions of the genome as detected by WGS. The right pie chart indicates the distribution of mutant types in coding region detected by WGS and WES. **(c)** The median number of non-synonymous mutations per tumor in a variety of tumor types. The red text and red arrow indicates average number of non-synonymous mutations per tumor in our cohort. (TIFF 8108 kb) Additional file 6: Figure S3. Comparison of somatic mutations rates between stage I and stage III tumors. **(a)** Nonsynonymous somatic coding mutation rates do not correlate with stage progression. Box plots showing number of somatic mutations, number of protein-altering somatic mutations and number of somatic mutations in candidate driver genes in stage I and stage III patients. Mean ± S.D. are indicated on the plots. All reported *p*-values test. **(b)** Comparison of broad structural genome alterations between subtypes of stage III tumors. Analysis is based on absolute copy numbers. Whole-genome sequencing-based analyses reveal that CNVs of subtypes of stage III show no significant difference. **(c)** Significant, focally amplified (red, upper panel) and deleted (blue, bottom panel) regions are plotted along the genome. The line represents G-score with 0.1. (TIFF 11404 kb) Additional file 7: Figure S4. Circos plot of intra- and inter-chromosomal translocations in all 14 WGS set. (TIFF 5431 kb) Additional file 8: Table S4. Full list of mutation events identified in our cohort. (XLSX 11 kb) Additional file 9: Figure S5. Focally amplified (red) region containing *FAM84B* viewed by IGV in 104 cohort is plotted along the chromosome. (TIFF 11424 kb) Additional file 10: Figure S6. A two-dimensional map of genetic alterations in ESCC. The horizontal axis represents genome position from 0 to 250 Mb, and the vertical axis represents genomic alterations including significantly mutated genes and other genes with mutation frequency > 2 % along with each chromosome. Significantly mutated genes as determined by MutSigCV are labeled. Each gene is scaled by a relative position (0–1) on its chromosome; thus, its position on different chromosomes was normalized by its total length. The heights of each cone represent frequencies of mutated genes among 104 patients. (TIFF 4925 kb) Additional file 11: Table S5. Focal CNAs in WGS set. GISTIC analysis was performed with 1 M window instead of array probe to identify significantly copy number alteration. (XLSX 1241 kb) Additional file 12: Table S6. Summary of significantly mutated genes and their distributions in stage I and stage III tumors. **(a)** Significantly mutated genes identified by MutSigCV analytical method (FDR <0.2). **(b)** Summary of frequently mutated genes and their distributions in stage I and stage III tumors. (XLSX 1846 kb) Additional file 13: Table S7.
*NOTCH1* mutations identified in 36 of atypical hyperplasia tissues through targeted PCR-Sanger sequencing. (XLSX 266 kb) Additional file 14: Table S8. Statistically significant pathway with different frequency in stage I and stage III tumors (*P* < 0.05). (XLS 27 kb) Additional file 15: Figure S7. Comparison of SMGs identified in EAC and ESCC. The star means SMG in the specific cohort. (TIFF 3743 kb) Additional file 16: Table S9. Summary of clinical data of large-scale TMA (OD-CT-DgEso01-001, Shanghai Outdo Biotech). (XLSX 200 kb) Additional file 17: Figure S8. Large-scale TMA analyses depict FAM84B expression pattern in ESCC tissues. Detail information of cases was shown in Table S9. (XLS 686 kb)

## Abbreviations

BWA: Burrows-Wheeler Aligner
ESCC: Esophageal squamous cell carcinoma
FAM84B: Family with sequence similarity 84, member B
GISTIC: Genomic identification of significant targets in cancer
HNSCC: Head and neck squamous cell carcinoma
IGV: Integrative genomics viewer
SCNA: Somatic copy number alterations
SMG: Significantly mutated gene
SNV: Single nucleotide variation
TCS: Target capture sequencing
WES: Whole-exome sequencing
WGS: Whole-genome sequencing
