# Supplementary figures and images for: Genomic analyses reveal FAM84B and the NOTCH pathway are associated with the progression of esophageal squamous cell carcinoma
Source: Gigascience. 2016 Jan 11;5:1. doi: 10.1186/s13742-015-0107-0 (PMC4709967; doi:10.1186/s13742-015-0107-0)

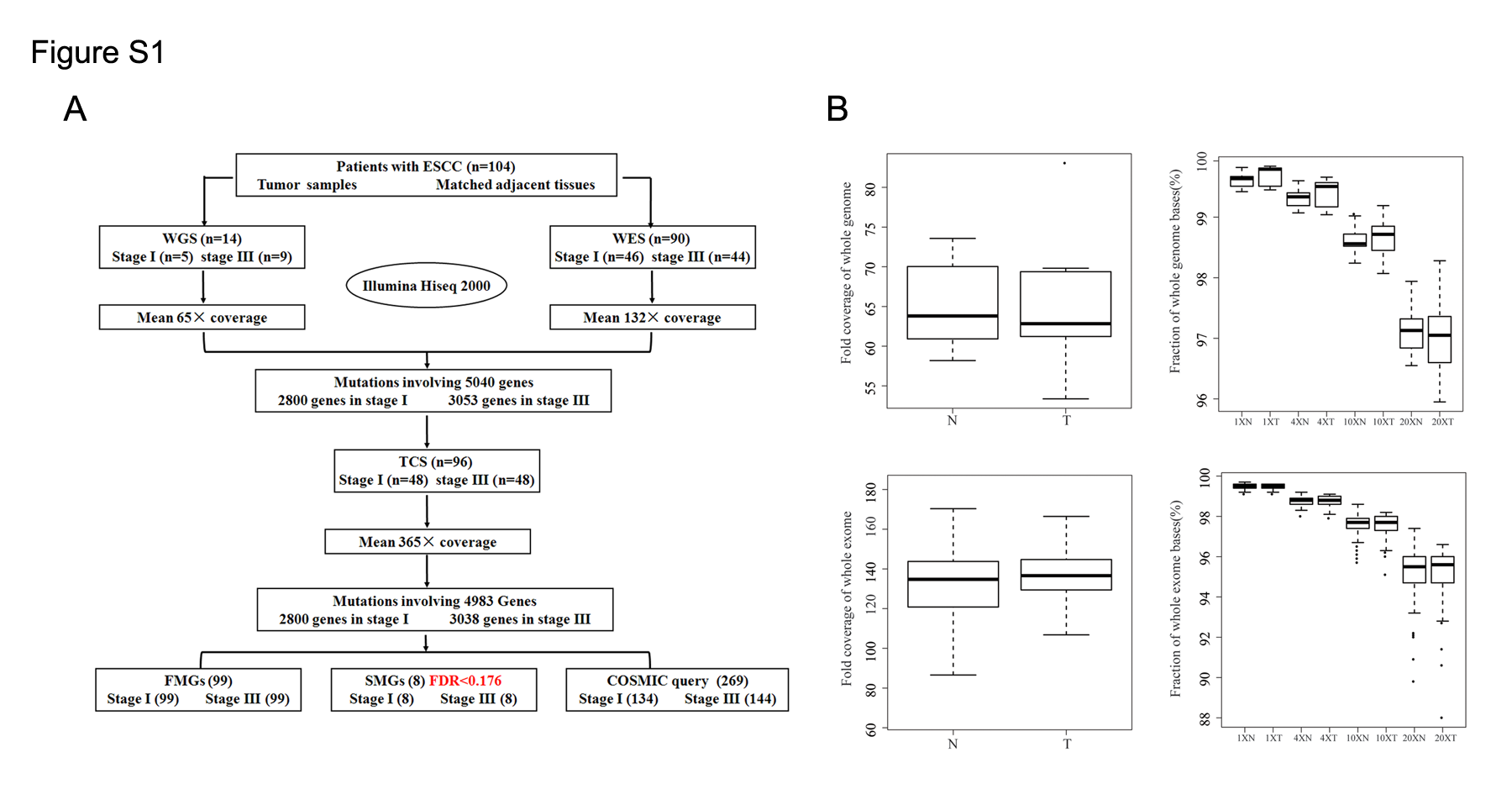

Supplement: Additional file 2: Figure S1. — The sequencing data processing pipeline and calculation of coverage. (a) Sequencing and analytical pipeline for determining somatic mutations in our cohort. (b) Fold coverage of whole genome and exome in the sequenced normal and tumor samples in ESCC. The upper panel: whole-genome sequencing set; the lower panel: whole-exome sequencing set; lfet: the box plot depicts the distribution of mean coverage; right: the box plot depicts the distribution of fraction of bases covered by at least 1 reads, 4 reads, 10 reads and 20 reads across the sequencing samples. N, normal samples; T, tumor samples; All samples were calculated with an average estimated tumor content of 40-50 %. (TIFF 4975 kb) [file 13742_2015_107_MOESM2_ESM.tiff]

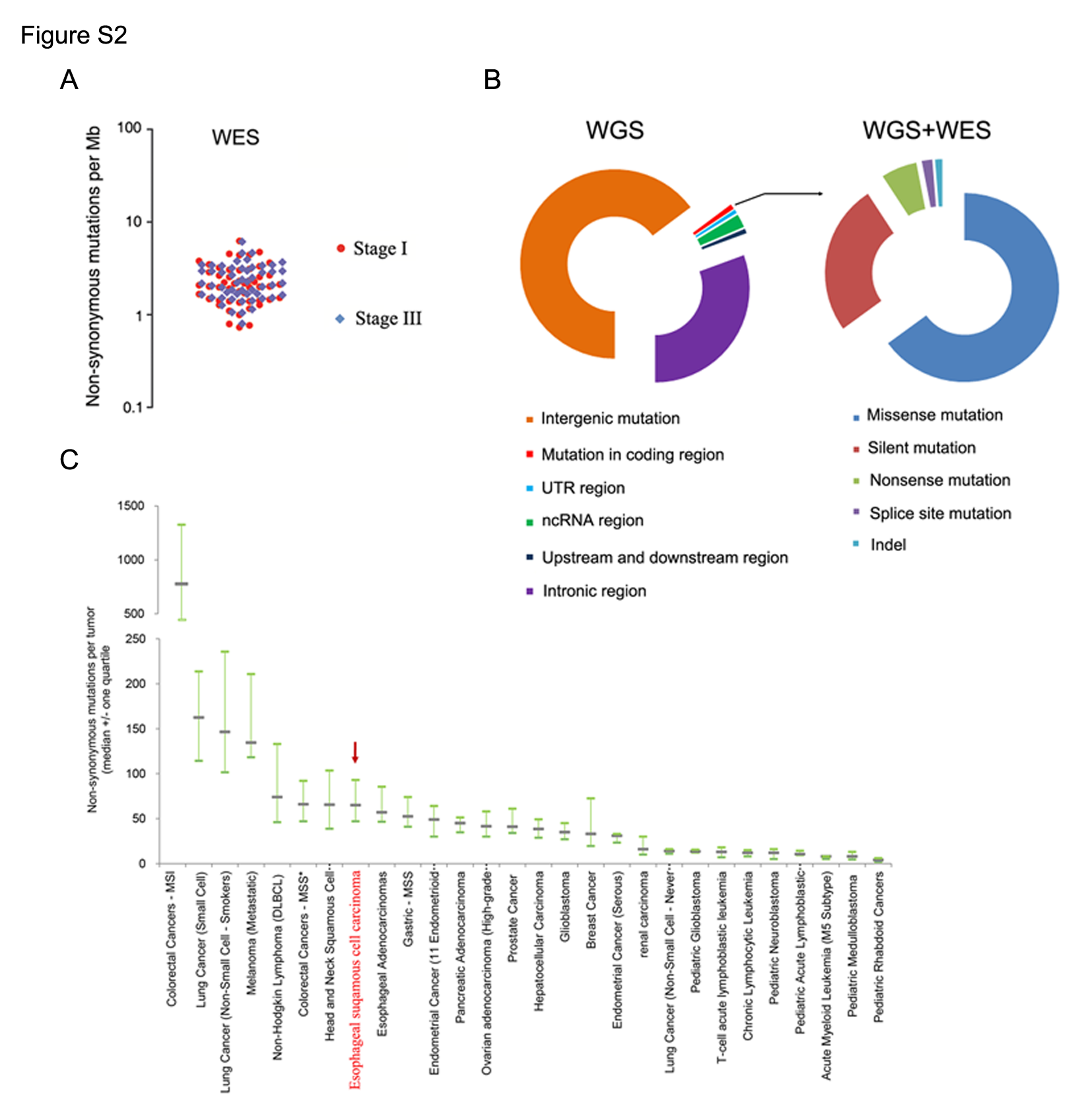

Supplement: Additional file 5: Figure S2. — Overview of mutations in ESCC and comparison with other tumor types. (a) Distribution of non-synonymous mutations identified in WES set. The red and blue spots represent mutations of stage I and stage III tumors, respectively. (b) The left pie chart shows the distribution of mutant regions of the genome as detected by WGS. The right pie chart indicates the distribution of mutant types in coding region detected by WGS and WES. (c) The median number of non-synonymous mutations per tumor in a variety of tumor types. The red text and red arrow indicates average number of non-synonymous mutations per tumor in our cohort. (TIFF 8108 kb) [file 13742_2015_107_MOESM5_ESM.tiff]

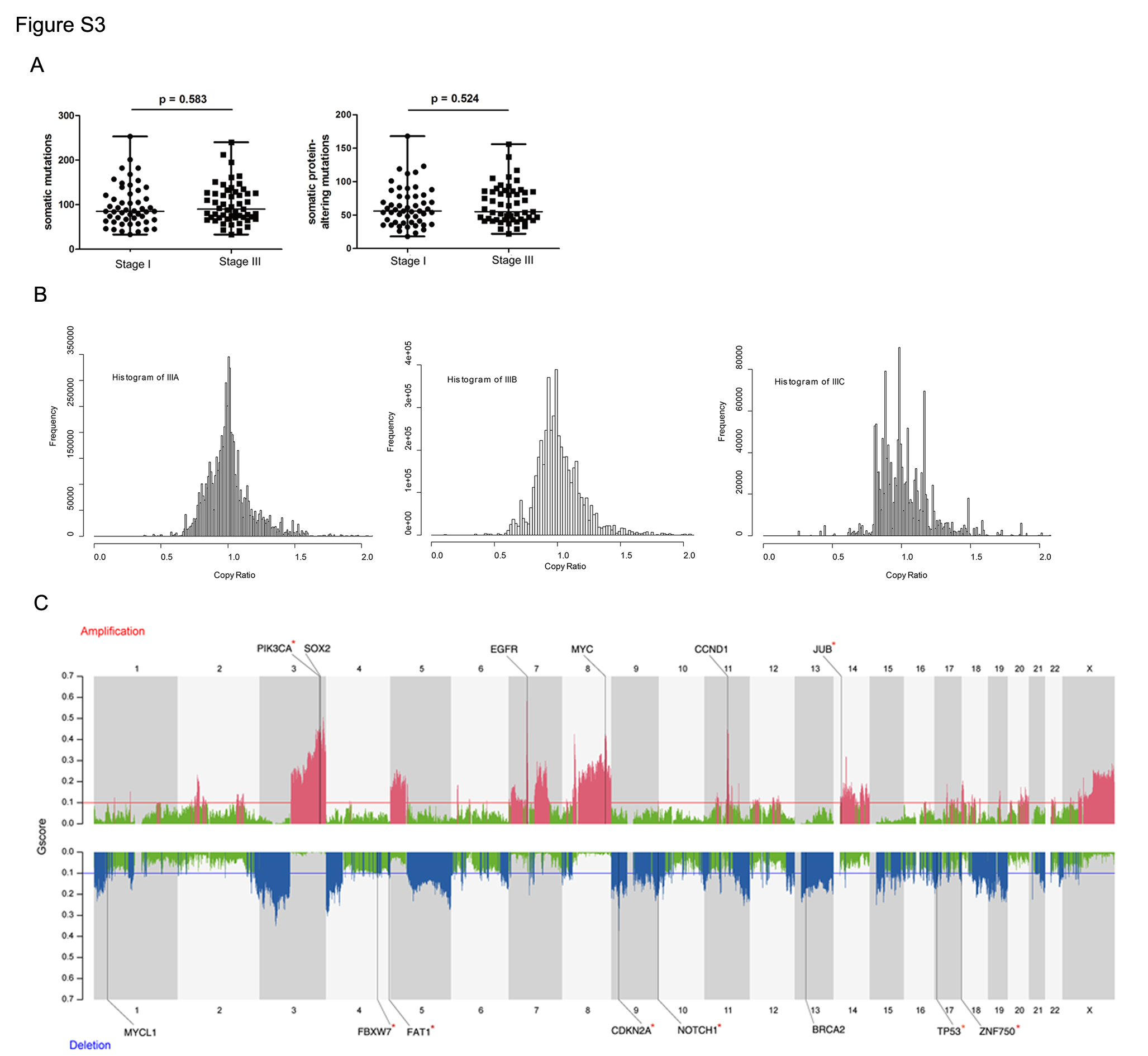

Supplement: Additional file 6: Figure S3. — Comparison of somatic mutations rates between stage I and stage III tumors. (a) Nonsynonymous somatic coding mutation rates do not correlate with stage progression. Box plots showing number of somatic mutations, number of protein-altering somatic mutations and number of somatic mutations in candidate driver genes in stage I and stage III patients. Mean ± S.D. are indicated on the plots. All reported p-values test. (b) Comparison of broad structural genome alterations between subtypes of stage III tumors. Analysis is based on absolute copy numbers. Whole-genome sequencing-based analyses reveal that CNVs of subtypes of stage III show no significant difference. (c) Significant, focally amplified (red, upper panel) and deleted (blue, bottom panel) regions are plotted along the genome. The line represents G-score with 0.1. (TIFF 11404 kb) [file 13742_2015_107_MOESM6_ESM.tiff]

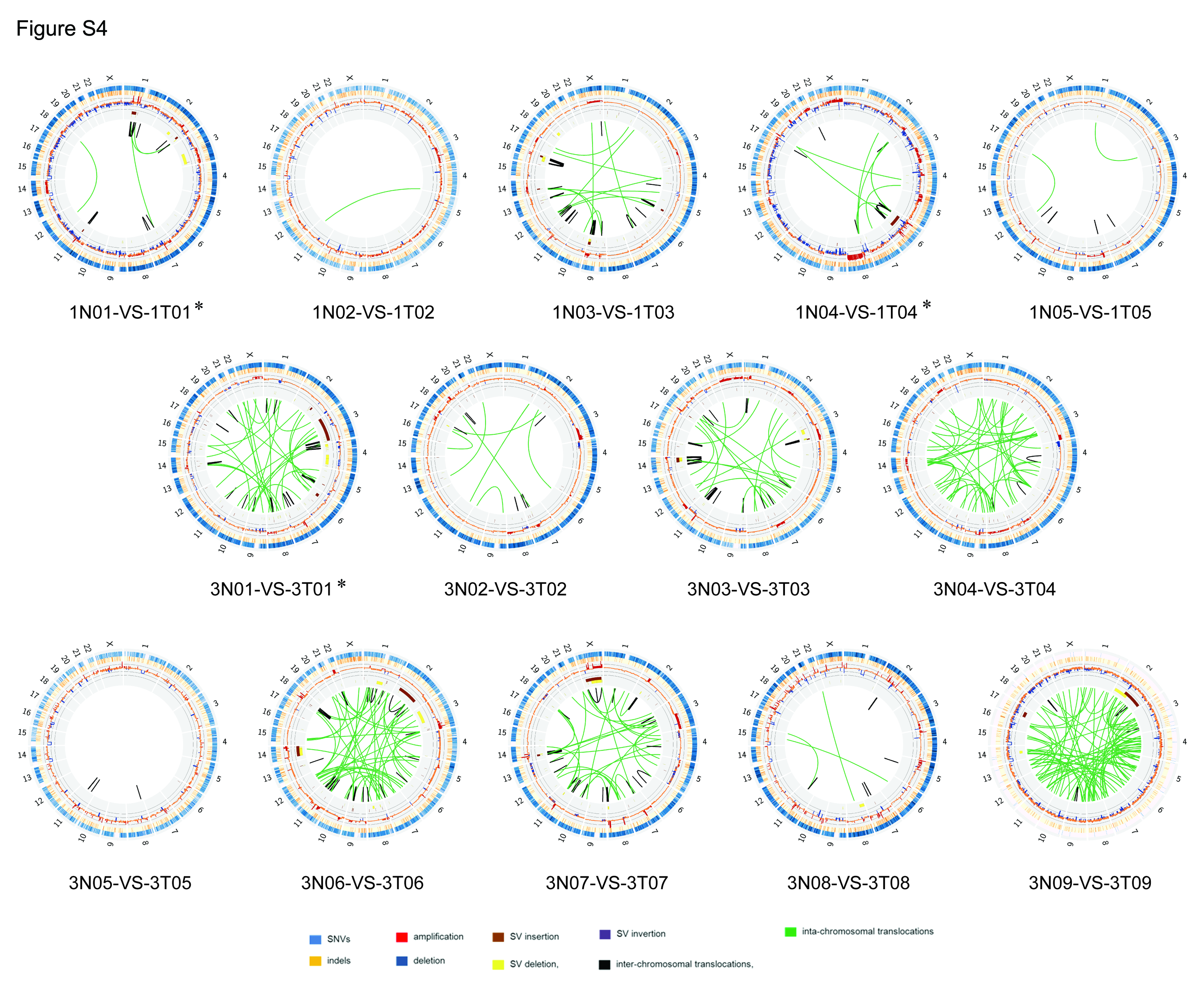

Supplement: Additional file 7: Figure S4. — Circos plot of intra- and inter-chromosomal translocations in all 14 WGS set. (TIFF 5431 kb) [file 13742_2015_107_MOESM7_ESM.tiff]

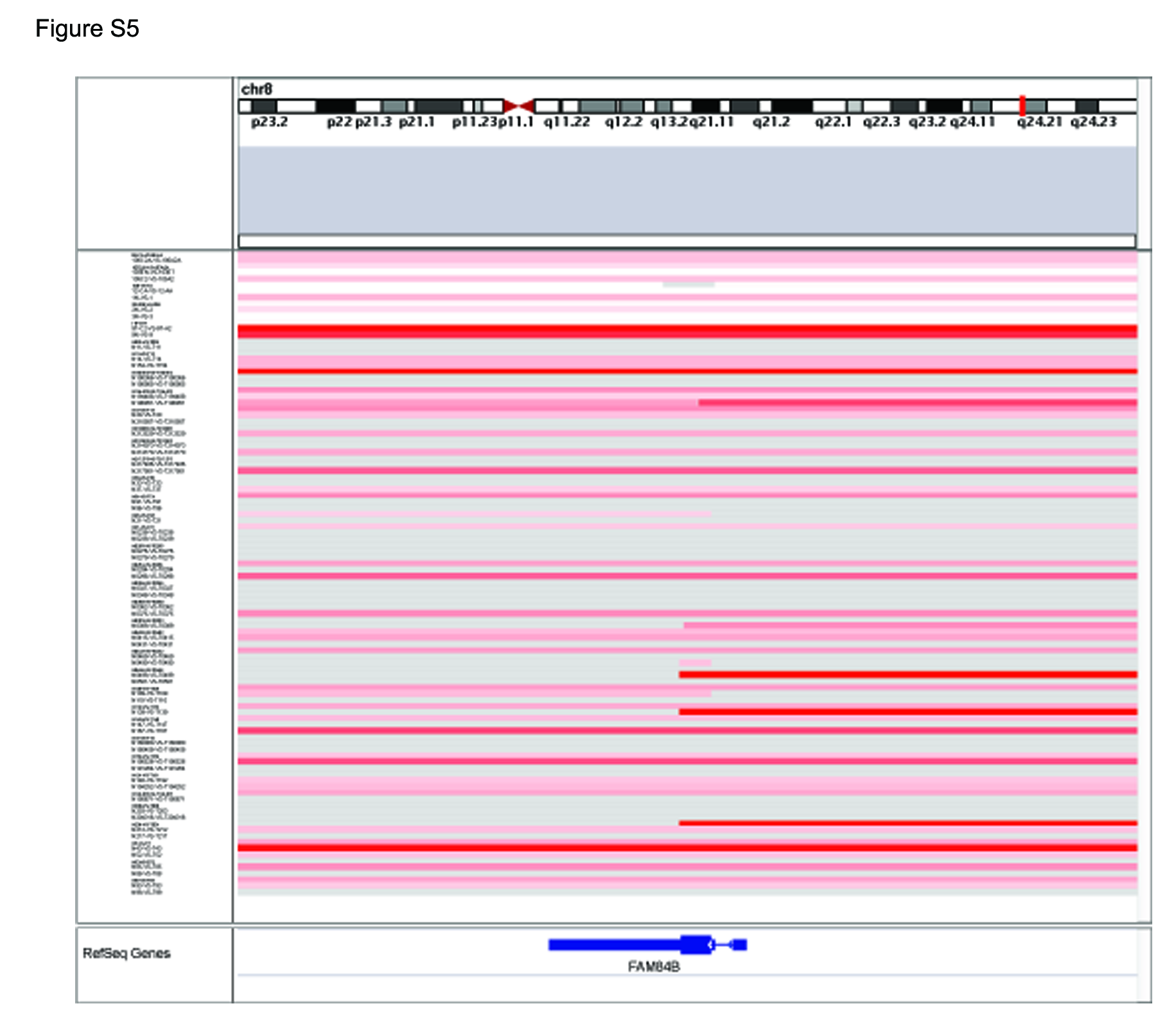

Supplement: Additional file 9: Figure S5. — Focally amplified (red) region containing FAM84B viewed by IGV in 104 cohort is plotted along the chromosome. (TIFF 11424 kb) [file 13742_2015_107_MOESM9_ESM.tiff]

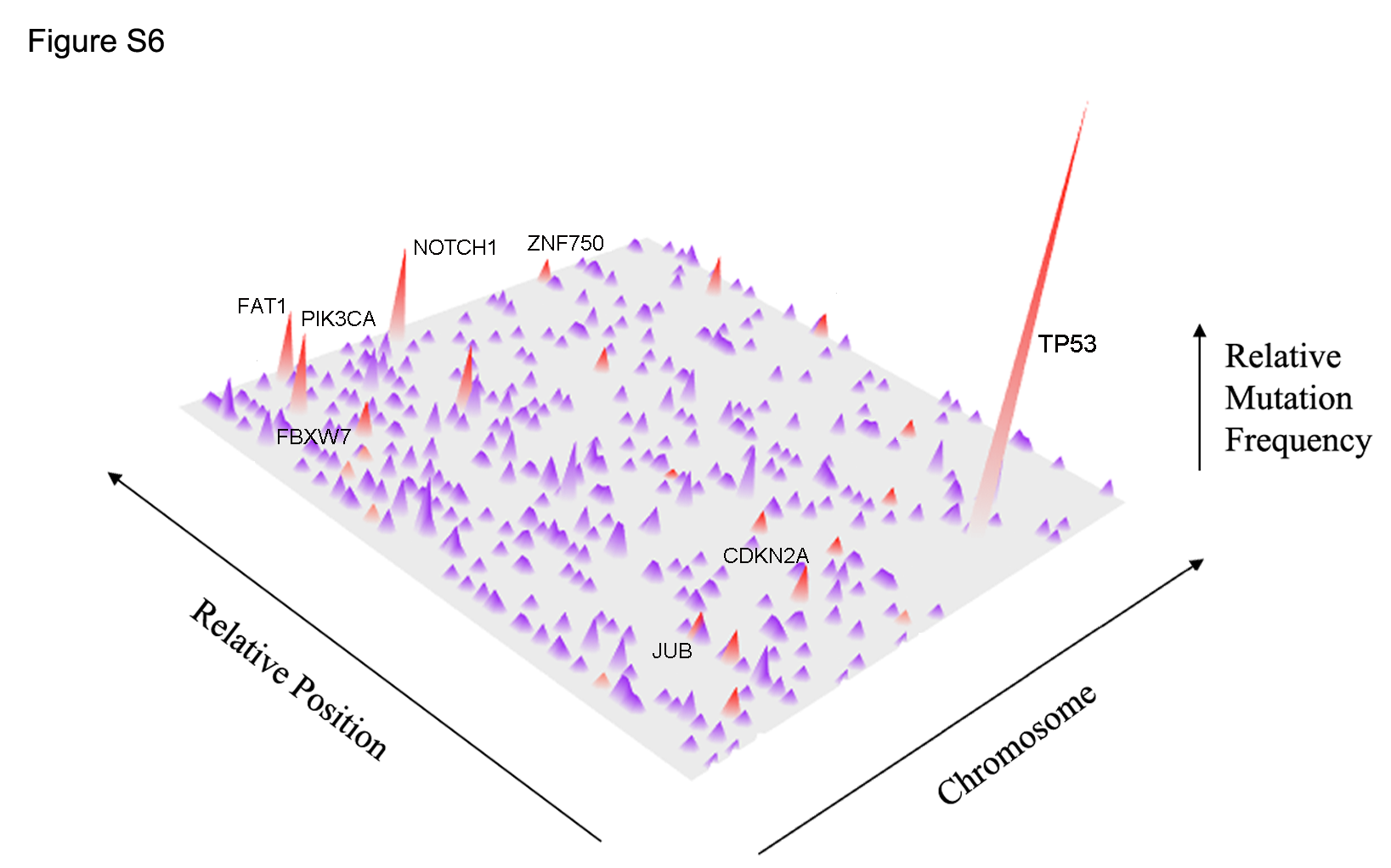

Supplement: Additional file 10: Figure S6. — A two-dimensional map of genetic alterations in ESCC. The horizontal axis represents genome position from 0 to 250 Mb, and the vertical axis represents genomic alterations including significantly mutated genes and other genes with mutation frequency > 2 % along with each chromosome. Significantly mutated genes as determined by MutSigCV are labeled. Each gene is scaled by a relative position (0–1) on its chromosome; thus, its position on different chromosomes was normalized by its total length. The heights of each cone represent frequencies of mutated genes among 104 patients. (TIFF 4925 kb) [file 13742_2015_107_MOESM10_ESM.tiff]

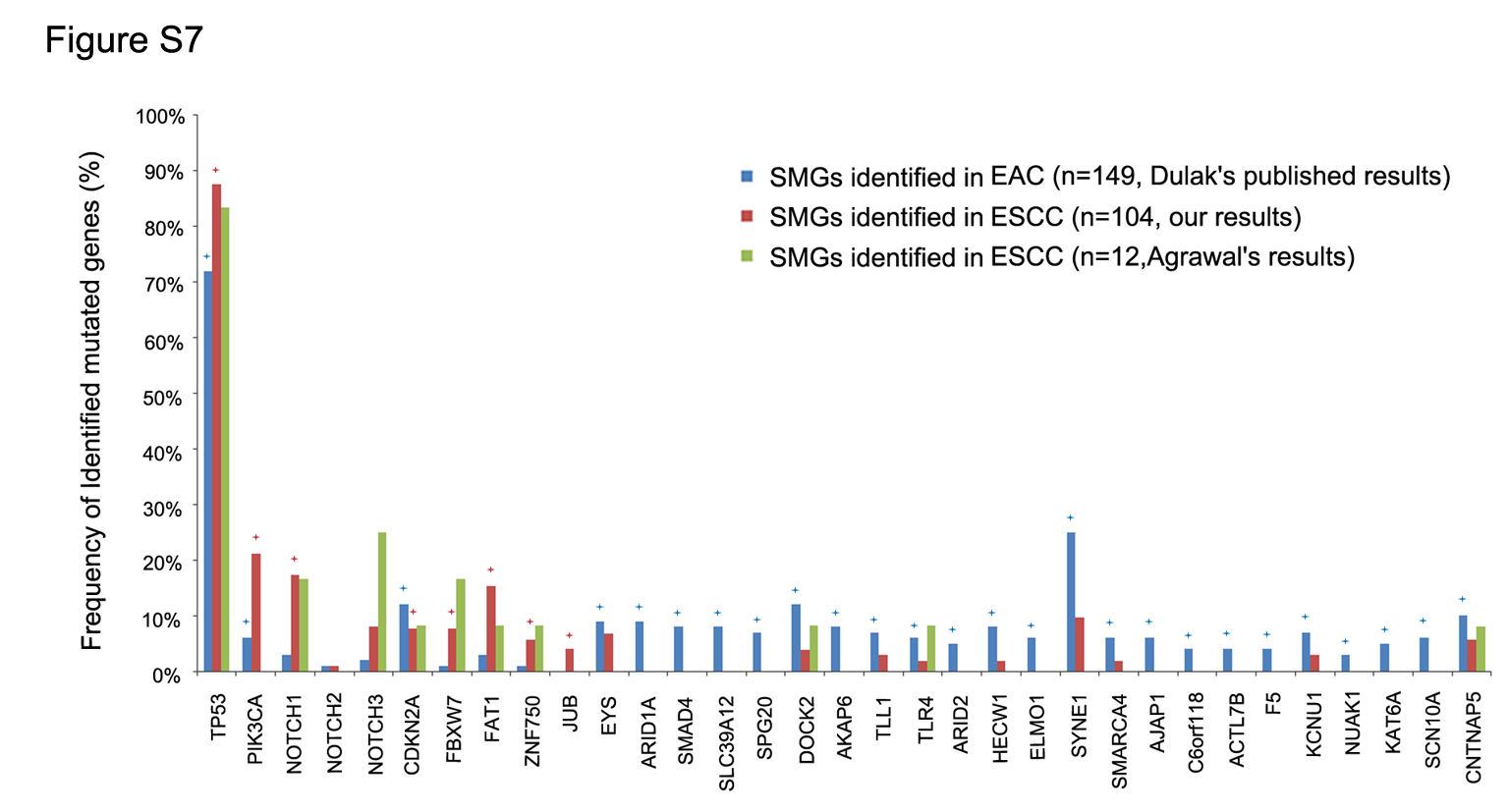

Supplement: Additional file 15: Figure S7. — Comparison of SMGs identified in EAC and ESCC. The star means SMG in the specific cohort. (TIFF 3743 kb) [file 13742_2015_107_MOESM15_ESM.tiff]
